# A Combined Elevation Angle and C/N0 Weighting Method for GNSS PPP on Xiaomi MI8 Smartphones

**DOI:** 10.3390/s22072804

**Published:** 2022-04-06

**Authors:** Yanjie Li, Changsheng Cai, Zhenyu Xu

**Affiliations:** School of Geosciences and Info-Physics, Central South University, Changsha 410083, China; lee_yanjie@163.com (Y.L.); northernbaldy@163.com (Z.X.)

**Keywords:** GNSS, smartphone, precise point positioning, elevation angle, C/N0

## Abstract

Traditionally, an elevation-angle-dependent weighting method is usually used for Global Navigation Satellite System (GNSS) positioning with a geodetic receiver. As smartphones adopt linearly polarized antenna and low-cost GNSS chips, different GNSS observation properties are exhibited. As a result, a carrier-to-noise ratio (C/N0)-dependent weighting method is mostly used for smartphone-based GNSS positioning. However, the C/N0 is subject to the effects of the observation environment, resulting in an unstable observation weight. In this study, we propose a combined elevation angle and C/N0 weighting method for smartphone-based GNSS precise point positioning (PPP) by normalizing the C/N0-derived variances to the scale of the elevation-angle-derived variances. The proposed weighting method is validated in two kinematic PPP tests with different satellite visibility conditions. Compared with the elevation-angle-only and C/N0-only weighting methods, the combined weighting method can effectively enhance the smartphone-based PPP accuracy in a three-dimensional position by 22.7% and 24.2% in an open-sky area, and by 52.0% and 26.0% in a constrained visibility area, respectively.

## 1. Introduction

The Global Navigation Satellite System (GNSS)-based navigation and positioning applications on smartphones have greatly aided our lives. The demand for precise positioning services on smartphones is increasing daily and attracting great attention in the GNSS community. For the GNSS positioning, its stochastic model is vital to determine the positioning accuracy when estimating the position parameters [1]. For geodetic-type GNSS receivers, an elevation-angle-dependent weighting method is usually used due to a strong correlation between the elevation angle and observation noise [2]. However, for smartphone-based GNSS receivers, due to hardware condition limitations and the complexity of the application environment, their observations exhibit different properties, such as large observation noise, drastic C/N0 variations and being prone to suffer from the multipath effect and even observation outages [3,4,5]. The observation noise and multipath effect on smartphones are more correlative to the carrier-to-noise ratio (C/N0) than the satellite elevation angle [6,7,8]. Consequently, the C/N0-dependent weighting method is mostly used in the smartphone-based GNSS positioning.

Several scholars evaluated the precise point positioning (PPP) performance on smartphones using different weighting methods. Chen et al. [9] used a Xiaomi MI8 smartphone to perform single-frequency PPP in a static mode based on an elevation-angle-dependent stochastic model, and the results showed that the average root mean square (RMS) of positioning errors can reach 0.81 m and 1.65 m in the horizontal and vertical directions, respectively. Furthermore, Wu et al. [10] used an elevation-angle-dependent stochastic model for static dual-frequency PPP on the Xiaomi MI8 smartphone and achieved a converged position accuracy of 0.22 m, 0.04 m and 0.11 m in the east, north and up directions, respectively. Nevertheless, the positioning accuracy decreased to 3–4 m in the kinematic mode due to insufficient dual-frequency observations. In addition, using the Xiaomi MI8 smartphone, Shinghal and Bisnath [11] utilized the C/N0-dependent stochastic model to improve the static PPP three-dimensional (3D) accuracy by about 27.0% over the elevation-angle-dependent stochastic model. Zhu et al. [12] used a Huawei Mate 30 smartphone to test the PPP in a kinematic mode based on the C/N0-dependent stochastic model to achieve a positioning accuracy of 0.93 m, 0.62 m and 2.17 m in the east, north and up directions, respectively; the accuracy was improved by about 26.2%, 20.5% and 20.4% when compared with the elevation-angle-dependent stochastic model. From the existing research, the advantage of the C/N0-dependent stochastic model is obvious for smartphone-based GNSS positioning. Although the C/N0-dependent weighting methods are mostly used in the smartphone-based GNSS positioning, the C/N0 is subject to the effects of the observation environment and severely fluctuates in the dynamic process. As a result, the C/N0-derived observation weights are prone to instability. Recently, a combined elevation angle and C/N0 weighting method was applied to smartphone-based kinematic PPP by simply dividing the C/N0-derived variances by the elevation-angle-derived variances. A sub-meter positioning accuracy can be achieved based on the combined weighting method [13]. However, its performance was not compared with the other weighting methods and thus its advantage is unclear.

In this study, a new combined elevation angle and C/N0 weighting method is proposed for smartphone-based PPP by normalizing the C/N0-derived variances to the scale of the elevation-angle-derived variances. The performance of the proposed weighting method is compared with the elevation-angle-only and C/N0-only weighting methods in two kinematic smartphone-based PPP tests. The paper is outlined as follows: firstly, the correlations among the code multipath and noise (CMN), C/N0 and elevation angle are analyzed; secondly, a combined elevation angle and C/N0 weighting method for the smartphone-based PPP is presented; and finally, the proposed weighting method is evaluated in two kinematic PPP experiments.

## 2. Correlation of CMN, Elevation Angle and C/N0

**Figure 1:**
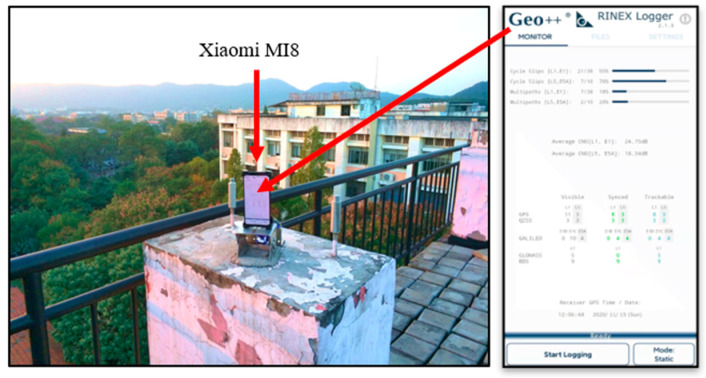
Smartphone data collection in an open sky area on 15 November 2020.

To analyze the correlation of the CMN with the elevation angle and C/N0, a Xiaomi MI8 smartphone equipped with a BCM47755 GNSS chip and a linearly polarized antenna is used as the experimental device. The Xiaomi MI8 smartphone is the first-released dual-frequency Android smartphone. Figure 1 shows the GNSS observation on the roof of the Mining Building at Central South University, China on 15 November 2020. The smartphone application of GEO++ RINEX Logger 2.1.3 is used for the data collection [14]. The observation lasts about 5 h from GPS Time 9:00 to 14:00 with a sampling rate of 1 HZ. The Xiaomi MI8 smartphone can receive dual-frequency GPS L1/L5 signals and Galileo E1/E5a signals, as well as single-frequency GLONASS G1 and BDS B1 signals.

The CMN is a major error source on smartphones due to the built-in linear polarization antenna [3,4]. The multipath effect at an epoch ($M_{i}$) can be estimated using the multipath combination below [15,16]:

(1) $$M_{i}=P_{i}-\frac{f_{i}^{2}+f_{j}^{2}}{f_{i}^{2}-f_{j}^{2}}\varphi_{i}\lambda_{i}+\frac{2f_{j}^{2}}{f_{i}^{2}-f_{j}^{2}}\varphi_{j}\lambda_{j}$$

where i and j (i≠j) denote two different frequencies. P is the pseudorange observation. φ is the carrier phase observation. λ is the wavelength at the corresponding frequency, f. $M_{i}$ contains multipath effect, code noise, ambiguity term and hardware delay biases. The latter two items are stable and thus can be obtained by calculating the mean value of $M_{i}$ at a certain number of epochs free of cycle slips [17], which is denoted as ${\bar{M}}_{i}$. Therefore, the CMN can be derived as:

(2) $$CMN_{i}=M_{i}-{\bar{M}}_{i}$$

**Figure 2:**
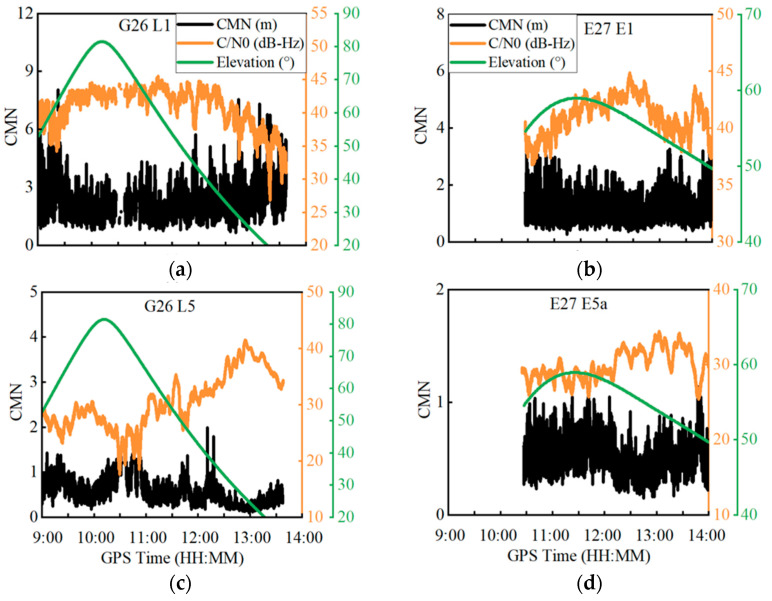
Time series of the code multipath and noise (CMN), carrier-to-noise ratio (C/N0) and elevation angle for G26 satellite at L1/L5 frequencies and E27 satellite at E1/E5a frequencies.

The correlation of the CMN to the satellite elevation angle and C/N0 was examined; Figure 2 shows the time series of the CMN, elevation angle and C/N0 for the GPS G26 and Galileo E27 satellites at both the L1/E1 and L5/E5a frequencies. In terms of the CMN amplitudes, the G26 satellite has a stronger CMN than the E27 satellite, and both satellites at the L1/E1 frequencies have a stronger CMN than the L5/E5a frequencies. These results are in line with the conclusions drawn by [3], which indicate that the Galileo satellites have a better CMN suppression ability than the GPS satellites and that the L5/E5a signals have a stronger CMN suppression ability than the L1/E1 signals. In addition, the CMN has a strong negative correlation with the C/N0 at both the L1/E1 and L5/E5a frequencies. When the CMN dramatically changes, the corresponding C/N0 vastly varies by over 10 dB-Hz. In contrast, the correlation with the elevation angle is relatively poor at the L1/E1 frequencies. In spite of this, their correlation is still obvious. When the elevation angle decreases, the CMN increases. However, at the L5/E5a frequencies, no obvious correlation is found between the CMN and the elevation angle, which might be caused by the irregular gain pattern of smartphones [18].

**Figure 3:**
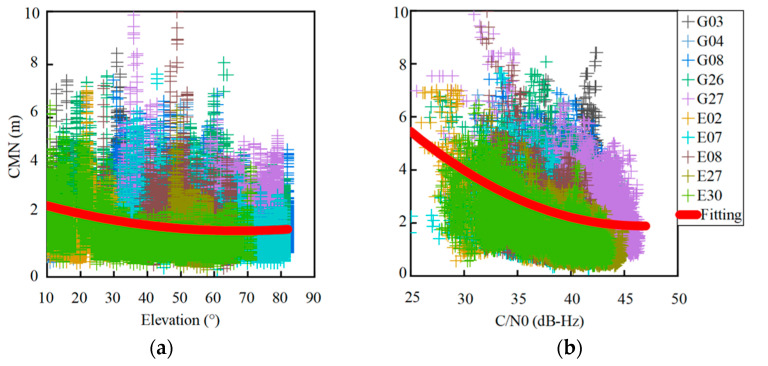
CMN of dual-frequency GPS and Galileo satellites against elevation angles (**a**) and C/N0 (**b**) at L1/E1 frequencies.

In order to further analyze the correlation of the CMN to the satellite elevation angle and C/N0 at the L1/E1 frequencies, the CMN for all dual-frequency GPS and Galileo satellites is plotted against the elevation angle and the C/N0, respectively, as shown in Figure 3. A second-order polynomial fitting curve is also plotted to reflect the variation trend in panels (a) and (b). The fitting curves illustrate that the CMNs have a negative correlation with the elevation angle and the C/N0. Their correlation coefficients are −0.20 and −0.37, respectively.

**Figure 4:**
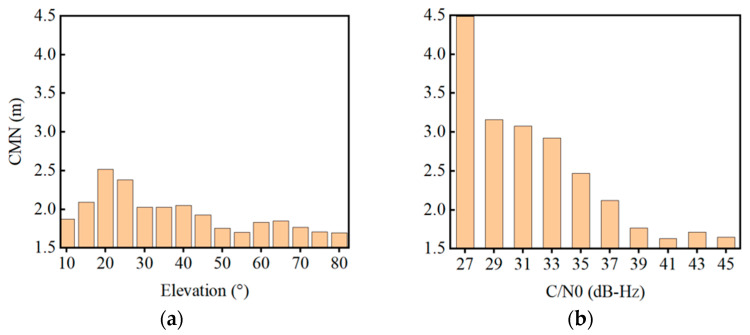
CMN mean value statistics against the elevation angle (**a**) and C/N0 (**b**).

Figure 4 shows the CMN mean value statistics against the elevation angle per five degrees and against the C/N0 per 2 dB-Hz. In summary, it is obvious that the CMNs have a negative correlation with the elevation angle and C/N0. In contrast, their correlation to the C/N0 is stronger than the elevation angle.

**Figure 5:**
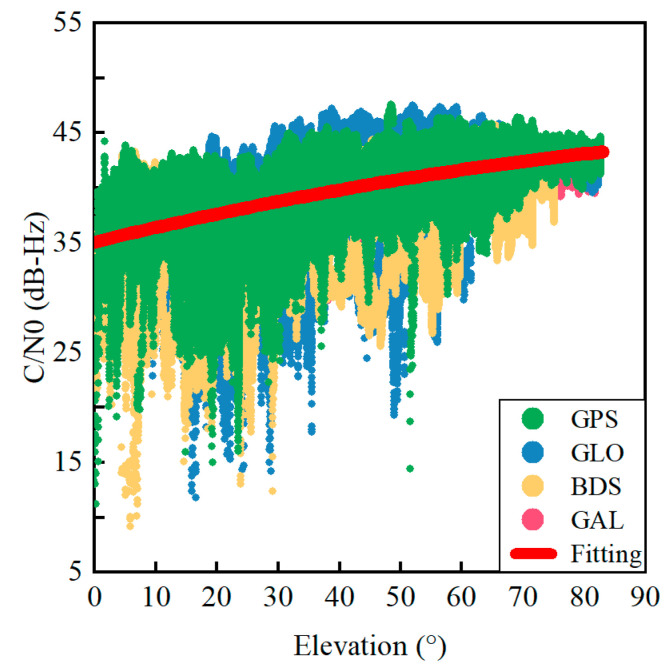
C/N0 of quad-constellation GNSS against elevation angles at L1/G1/B1/E1 frequencies.

Figure 5 shows the relationship between the C/N0 and elevation angle for quad-constellation GNSS at L1/G1/B1/E1 frequencies. A second-order polynomial fitting curve is employed to reflect the correlation between the C/N0 and elevation angle. As can be seen, their correlation is obvious since the C/N0 increases as the elevation angle increases.

## 3. Combined Elevation Angle and C/N0 Weighting Method 

Generally, the observation weight matrix W can be depicted as [19]:

(3) $$W=\mathrm{diag}\left(\sigma_{1}^{-2},\sigma_{2}^{-2},\ldots ,\sigma_{m}^{-2}\right)$$

where m is the number of observations; $\sigma^{2}$ is the observation variance, which can be expressed as:

(4) $$\sigma^{2}=\sigma_{obs}^{2}+\sigma_{eph}^{2}+\sigma_{ion}^{2}+\sigma_{trop}^{2}$$

where $\sigma_{obs}^{2}$ is the receiver-related variance term and $\sigma_{eph}^{2}$, $\sigma_{ion}^{2}$ and $\sigma_{trop}^{2}$ are the variances of the satellite orbit and clock error, ionosphere correction model error and troposphere correction model error, respectively. In this study, we mainly focus on the effect of the receiver-related variance term on the observation weight for smartphone-based GNSS positioning. Therefore, the following mentioned observation variance only refers to the receiver-related variance term $\sigma_{obs}^{2}$.

The traditional observation weighting methods for smartphone-based GNSS positioning mainly rely on the elevation angle or C/N0 [20]. The commonly used elevation-angle-dependent weighting model is depicted as [21,22]:

(5) $$\sigma_{\mathrm{ele}}^{2}{=\sigma }_{0}^{2}{\;/\sin}^{2}\left(\mathrm{ele}\right)$$

where $\sigma_{\mathrm{ele}}^{2}$ is the observation variance; $\sigma_{0}^{2}$ is a constant variance value; sin is the sine function and ele is the satellite elevation angle. 

A commonly used C/N0 weighting model is expressed as [23,24]:

(6) $$\sigma_{C/N0}^{2}{=\sigma }_{0}^{2}{\;\times \;10}^{\frac{\max(\mathrm{MAX}\;-\;C/N0,\;0)}{10}}$$

where $\sigma_{C/N0}^{2}$ is the observation variance; max is the maximum function and MAX is a preset maximum C/N0. 

To jointly use the satellite elevation angle and C/N0 information to determine the observation weight, we propose a combined elevation angle and C/N0 weighting method by normalizing the C/N0-derived variances to the scale of the elevation-angle-derived variances. The combined weighting method is established following the steps below. 

Firstly, the correlation between the satellite elevation angle and the C/N0 at the L1/G1/B1/E1 frequencies is obvious, as analyzed in Section 2. Thus, a second-order polynomial function is employed to fit the C/N0 against the satellite elevation angle, as depicted below:

(7) $$C/N0\_\mathrm{cal}=a+b\times \mathrm{ele}+c\times {\mathrm{ele}}^{2}$$

where C/N0_cal is the fitting C/N0 value and a, b and c are the fitting coefficients. According to the correlation between the satellite elevation angle and the C/N0 shown in Figure 5, the fitting coefficients of a, b and c can be taken as 35.0833 dB-Hz, 0.1365 dB-Hz/° and −0.0005 dB-Hz/(°)^2^, respectively and may be used as empirical values.

Secondly, the C/N0-derived variances are converted to the scale of the elevation-angle-derived variances to unify the variance scale of the two indicators. For the L1/G1/B1/E1 signals, the elevation-angle-derived variances are used as the main part to determine the observation weight, while the C/N0-derived variances, after subtracting the fitting-C/N0-derived variances, are used as a supplement for observation weight determination, as depicted in Equation (8). Such a weighting method can respond to the instantaneous variation in observation accuracy and simultaneously avoid obtaining unstable observation weights. For the L5/E5a signals, since the correlation between the CMN and the elevation angle is not obvious, as analyzed in Section 2, only the C/N0-derived variances are used to determine the observation weight. To keep consistent with the observation variances at the L1/G1/B1/E1 frequencies, the C/N0-derived variances are also normalized to the scale of the elevation-angle-derived variances:

(8) $$\{\begin{array}{l} \sigma_{L1}^{2}=\left|\sigma_{C/N0,\;L1}^{2}-\sigma_{C/N0\_\mathrm{cal}}^{2}\right|\;\times \;\frac{\sigma_{\mathrm{ele}\_\mathrm{MAX}}^{2}-\sigma_{\mathrm{ele}\_\mathrm{MIN}}^{2}}{\sigma_{C/N0\_\mathrm{MAX},\;L1}^{2}-\sigma_{C/N0\_\mathrm{MIN},\;L1}^{2}}+\sigma_{\mathrm{ele}}^{2}\\ \sigma_{L5}^{2}{=\sigma }_{C/N0,\;L5}^{2}\;\times \;\frac{\sigma_{\mathrm{ele}\_\mathrm{MAX}}^{2}-\sigma_{\mathrm{ele}\_\mathrm{MIN}}^{2}}{\sigma_{C/N0\_\mathrm{MAX},\;L5}^{2}-\sigma_{C/N0\_\mathrm{MIN},\;L5}^{2}} \end{array}$$

where $\sigma_{L1}^{2}$ and $\sigma_{L5}^{2}\;$ are the observation variances at the L1/G1/B1/E1 frequencies and the L5/E5a frequencies, respectively; C/N0 and C/N0_cal are the measured C/N0 and fitted C/N0, respectively; C/N0_MIN and C/N0_MAX are the minimum and maximum C/N0 values, which are set to 25 dB-Hz and 45 dB-Hz at the L1/G1/B1/E1 frequencies, and 20 dB-Hz and 40 dB-Hz at the L5/E5a frequencies, respectively and ele_MIN and ele_MAX are the minimum and maximum elevation angles, which are set to 10° and 90°, respectively.

The observation weight value is an inverse ratio of the observation variance. Once the observation variance is determined following Equation (8), the observation weight can be acquired.

## 4. Experimental Results and Discussion

In this section, the quad-constellation GPS/GLONASS/BDS/Galileo PPP processing strategy is provided in detail. Then, two kinematic smartphone-based GNSS PPP experiments are conducted to evaluate the combined elevation angle and C/N0 weighting method with comparisons to the conventional elevation-angle-only and C/N0-only weighting methods.

### 4.1. Quad-Constellation PPP Processing Strategy

An undifferenced and uncombined observation model is adopted for the smartphone-based quad-constellation PPP [25,26] to validate the proposed weighting method. The 2-Day predicted Global Ionospheric Map (GIM) products from the Chinese Academy of Sciences (CAS) are used as pseudo-observables to reduce the effect of ionospheric delay errors on the single-frequency observations [27,28]. The satellite PCO (Phase Center Offset) and PCV (Phase Center Variation) are corrected using the International GNSS Service (IGS) products, whereas the smartphone PCO is corrected using a recommended value from the reference [29]. The detailed PPP processing strategy is shown in Table 1.

The carrier phase observation precision is set to 0.01 m, whereas the pseudorange observation precision is set to 3 m [23]. In the quad-constellation PPP, the observation precision for different constellations and frequencies is different. We employ a four-order differential method [8,32] to analyze the observation precision for different constellations and frequencies. On the basis of a comparative analysis, the initial pseudorange observation weight ratio is set to 5:1:7:7 for GPS/GLONASS/BDS/Galileo at the L1/G1/B1/E1 frequencies and 1:1 for GPS/Galileo at the L5/E5a frequencies. Since the carrier phase observation precision is similar, an identical weight strategy is used for the quad-constellation carrier phase observations at both frequencies. For observations at the L1/E1 and L5/E5a frequencies, their weight ratio is set to 1:2 for pseudorange and 2:1 for carrier phase. 

### 4.2. Kinematic Experiment in an Open Sky Environment

**Figure 6:**
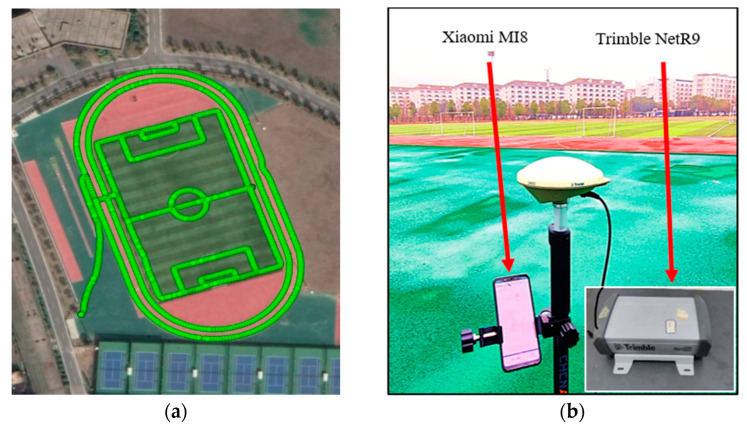
Kinematic experimental trajectory (**a**) and equipment setup (**b**) on an open-sky playground.

A kinematic GNSS experiment was conducted using the Xiaomi MI8 smartphone on an open playground of Central South University, China on 2 December 2021. Its trajectory and experiment equipment are shown in Figure 6. A Trimble NetR9 GNSS receiver with a geodetic antenna is employed to acquire the precise kinematic position for use as a benchmark value. The horizontal distance between the geodetic-type antenna and the smartphone is less than one decimeter. The experimental platform was lifted by a pedestrian over the head. A base station is set up at about one kilometer away from the rover station. Thus, the position of the geodetic receiver at the rover station can be precisely determined by the real-time kinematic (RTK) technique. The entire data collection lasts about 24 min, with a sampling rate of 1 HZ. The elevation mask is set to 10 degrees.

**Figure 7:**
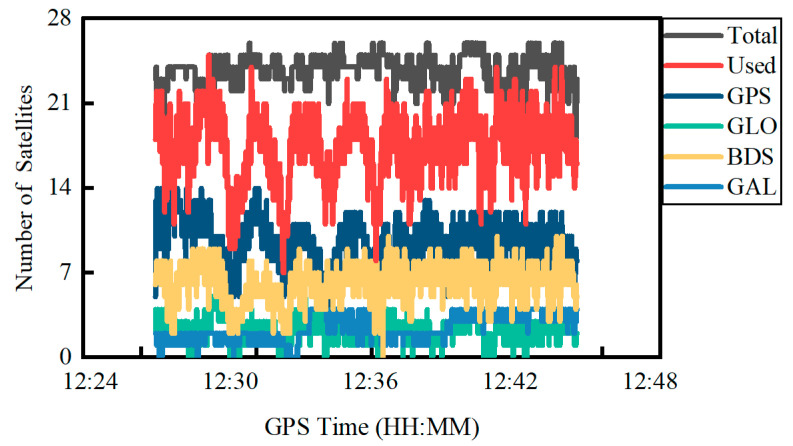
Number of satellites for quad-constellations on 2 December 2021.

Figure 7 shows the total number of satellites received, the total number of satellites used and the number of satellites for different constellations of GPS, GLONASS, BDS and Galileo in the PPP. The number of GPS and BDS satellites observed is obviously more than GLONASS and Galileo. At the same time, the total number of satellites received is 23.8 on average, which is apparently less than the Trimble NetR9 receiver at an average number of 36.0. In contrast, the total number of satellites used for PPP on the Xiaomi MI8 smartphone is only 17.5 on average with an STD of 2.8 due to a lack of parts of observations.

**Figure 8:**
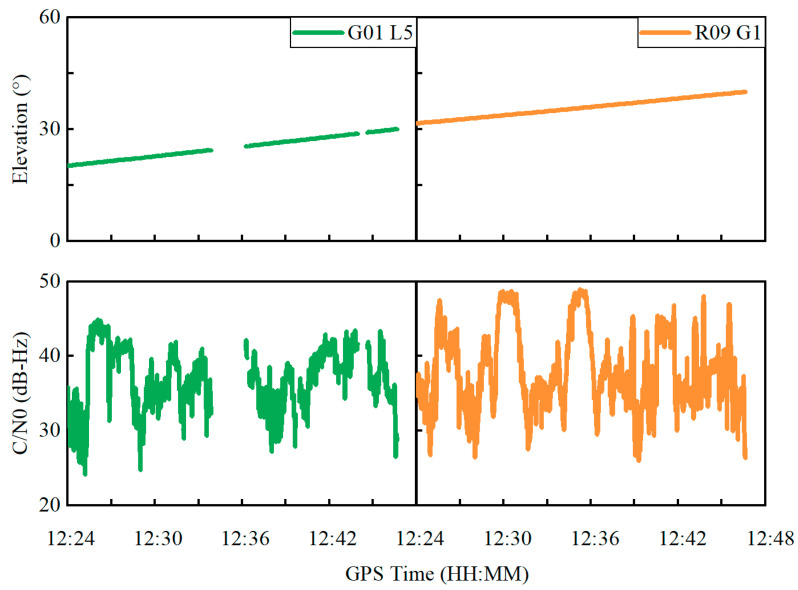
Elevation angle and C/N0 variations for GPS G01 satellite at L5 frequency and GLONASS R09 satellite at G1 frequency.

**Figure 9:**
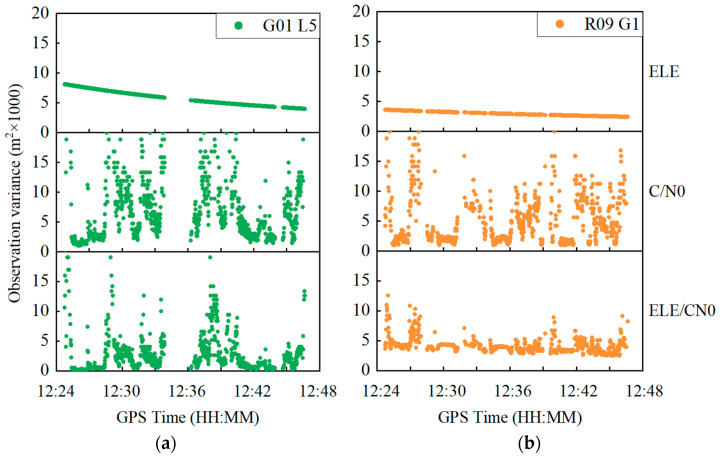
Carrier phase observation variances for GPS G01 satellite at L5 frequency (**a**) and GLONASS R09 satellite at G1 frequency (**b**). ELE, C/N0 and ELE/CN0 represent weighting scenarios of elevation-angle-only, C/N0-only and combined elevation angle and C/N0, respectively.

Taking the GPS G01 satellite at the L5 frequency and the GLONASS R09 satellite at the G1 frequency as examples, Figure 8 depicts the variations in elevation angle and C/N0 in the time domain, which shows that the elevation angle steadily changes for both satellites. In contrast, the C/N0 severely fluctuates with a range exceeding 10 dB-Hz for both satellites, which suggests that the C/N0 is prone to be affected by the outer environment due to a polarized antenna embedded inside the smartphone. Figure 9 shows the observation variances derived from Equations (5), (6) and (8) for the GPS G01 at the L5 frequency and the GLONASS R09 at the G1 frequency. The observation variances are displayed in the top, middle and bottom panels, derived from three different weighting scenarios, namely elevation-angle-only, C/N0-only and combined elevation angle and C/N0. As can be seen, the C/N0-derived observation variances severely vary, whereas the observation variances derived from the combined elevation angle and C/N0 are more stable.

**Figure 10:**
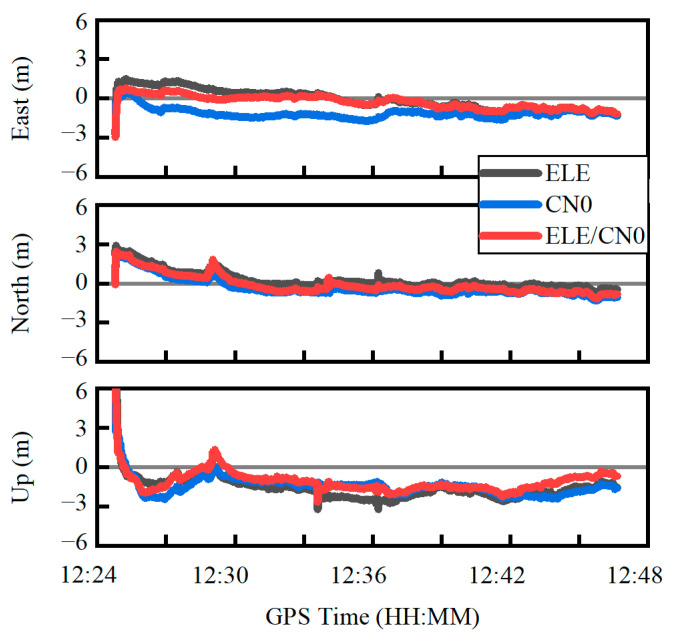
Quad-constellation PPP errors using three different weighting scenarios. ELE, C/N0 and ELE/CN0 represent weighting scenarios of elevation-angle-only, C/N0-only and combined elevation angle and C/N0, respectively.

Figure 10 shows the positioning errors in the east, north and up components using the three weighting scenarios. It can be seen that the positioning error curve derived from the combined weighting method is closer to the zero axis, especially in the east direction. The RMS statistics of the positioning errors are provided in Table 2 in the east, north and up components as well as the three-dimensional (3D) direction. Based on the combined weighting scenario, the RMS values of the position errors are 0.55 m, 0.73 m and 1.35 m in the east, north and up components, respectively, which improve by about 25.7%, 2.7% and 26.2% over the elevation-angle-only weighting scenario, and by about 53.8%, 5.2% and 16.7% over the C/N0-only weighting scenario. For the 3D position, the improvement can reach 22.7% and 24.2% over the elevation-angle-only and C/N0-only weighting scenarios, respectively. The obtained PPP accuracy is comparable to the existing research [12,13].

### 4.3. Kinematic Experiment in Constrained Visibility Environment

**Figure 11:**
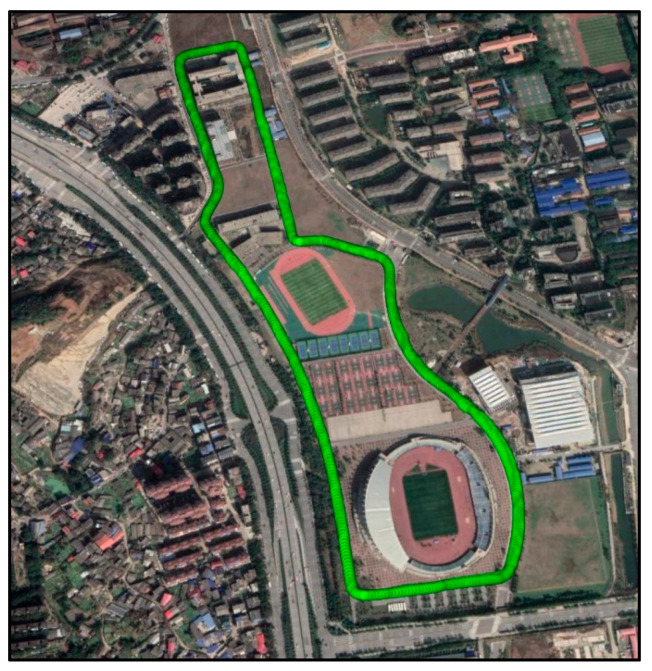
Kinematic experimental trajectory in a constrained satellite visibility environment.

A kinematic GNSS experiment was conducted using the Xiaomi MI8 smartphone in a constrained satellite visibility environment on 12 October 2021, on the new campus of Central South University, China. The satellite visibility is easily affected by the buildings and trees along its trajectory, as shown in Figure 11. The equipment setup is similar to the previous experiment shown in Figure 5. The experiment equipment was carried by an electric bicycle. The entire data collection lasts about half an hour with a sampling rate of 1 HZ. The elevation mask is set to 10 degrees.

**Figure 12:**
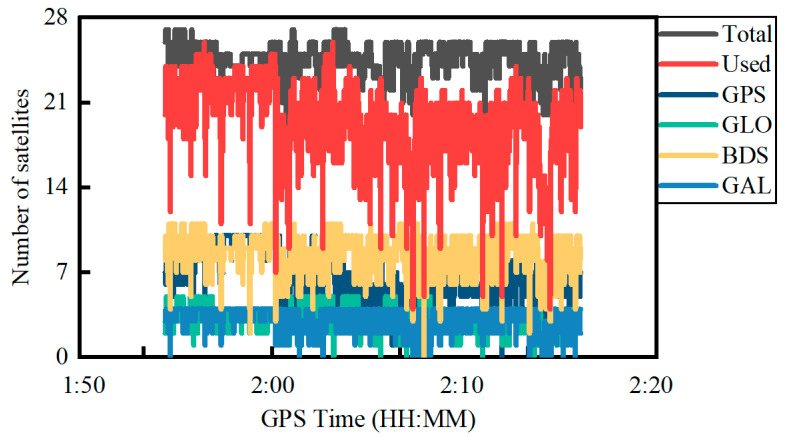
Number of satellites for quad-constellation GNSS on 12 October 2021.

Figure 12 shows the total number of satellites received, total number of satellites used and the number of satellites for different constellations of GPS, GLONASS, BDS and Galileo in the PPP. Similar to the previous experiment, the number of GPS and BDS satellites observed is more than the GLONASS and Galileo satellites. The total number of satellites received for the Trimble NetR9 receiver is less than that in the open area at an average of about six satellites due to the constrained satellite visibility environment. In contrast, the number of satellites used on the Xiaomi MI8 smartphone in the constrained environment is larger than that in the open area by an average of about two satellites, which indicates that the number of GNSS satellites observed on smartphones is unstable in different sessions. Furthermore, due to the observation environment influence, the number of satellites used in PPP significantly fluctuates.

**Figure 13:**
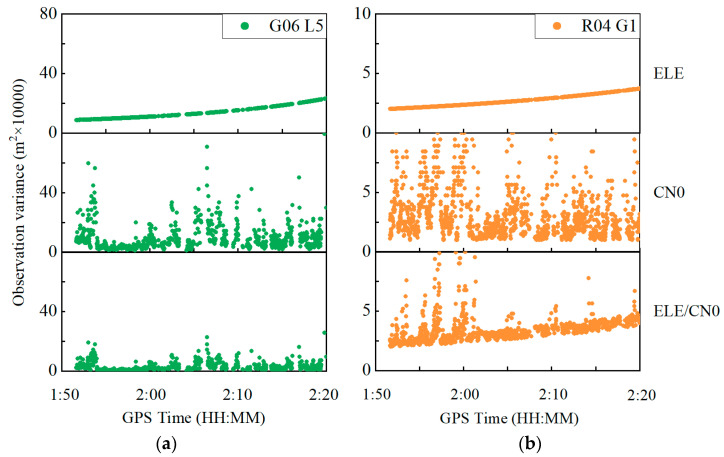
Carrier phase observation variances for GPS G06 satellite at L5 frequency (**a**) and GLONASS R04 satellite at G1 frequency (**b**).

Figure 13 shows the observation variances derived from Equations (5), (6) and (8) for the GPS G06 at the L5 frequency and the GLONASS R04 at the G1 frequency. The observation variances are displayed in the top, middle and bottom panels, derived from three different weighting scenarios, namely elevation-angle-only, C/N0-only and combined elevation angle and C/N0. Similar to the previous experiment, the C/N0-derived observation variances severely vary, while the observation variances derived from the combined elevation angle and C/N0 weighting scenarios are more stable.

**Figure 14:**
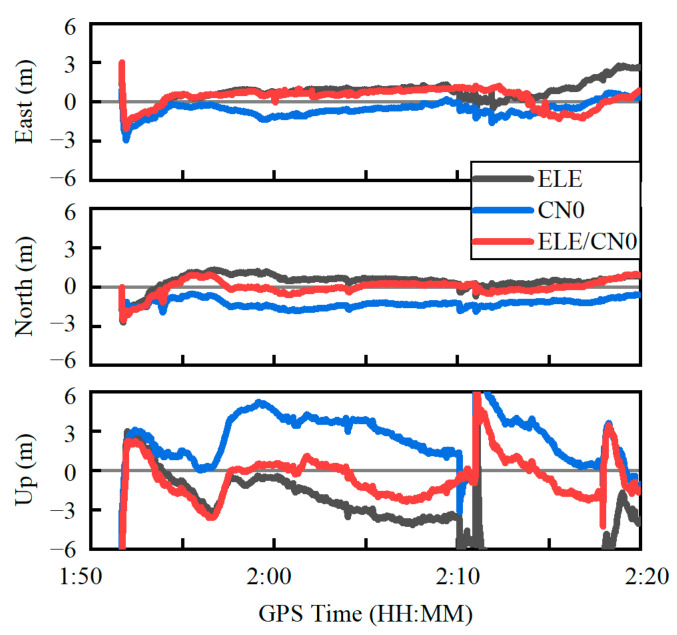
Quad-constellation PPP errors using three different weighting scenarios.

Figure 14 shows the positioning errors in the east, north and up components using the three weighting scenarios. It can be seen that the positioning error curves derived from the combined weighting scenario is much closer to the zero axis, especially in the north and up directions. The RMS statistics of the positioning errors are provided in Table 3. Based on the combined weighting scenario, the RMS values of the PPP errors are 1.00 m, 0.62 m and 2.22 m in the east, north and up components, respectively, which improve by about 19.3%, 12.7% and 55.9% over the elevation-angle-only weighting scenario in the east, north and up directions, respectively and by about 51.6% and 26.7% over the C/N0-only weighting scenario in the north and up components, respectively. For the 3D position, the corresponding improvement can reach 52.0% and 26.0%, respectively. In addition, it is more prone to reconvergence under the constrained satellite visibility condition, which is especially apparent in the up direction. The achieved PPP accuracy is comparable to the existing research [12,13].

## 5. Conclusions

The observation weighting method is vital for smartphone-based GNSS PPP. In this study, a combined elevation angle and C/N0 weighting method was proposed by taking advantage of the two indices. The obtained weight values were more stable than the C/N0-only derived weight values. Simultaneously, it also instantaneously responded to the observation accuracy variation. The proposed weighting method was fully evaluated in two GNSS PPP kinematic experiments using a Xiaomi MI8 smartphone.

Two kinematic PPP experiments were carried out in different satellite visibility conditions. Using the combined elevation angle and C/N0 weighting method, the RMS improvement of the 3D position for PPP reaches 22.7% and 24.2% over the elevation-angle-only and C/N0-only weighting methods, respectively in the open sky environment, and 52.0% and 26.0% over the elevation-angle-only and the C/N0-only weighting methods, respectively in the constrained visibility environment. It should be noted that all conclusions were drawn based on the Xiaomi MI8 GNSS kinematic experiments. Further smartphone experiments should be conducted in future research.

## Figures and Tables

**Table 1:** PPP processing strategy.

Items	Processing Strategies
Estimation method	Kalman filter
Constellations	GPS (L1, L5)/GLONASS (G1)/BDS (B1)/Galileo (E1, E5a)
Weighting scheme	Combined elevation angle and C/N0 weighting method
Satellite orbit and clock	Real-time precise satellite orbit and clock products from Centre National d’Etudes Spatiales (CNES) [30]
Ionospheric delay	Estimated as random walk process and GIM products are used as pseudo-observations
Tropospheric delay	Hydrostatic delay uses Saastamoinen model correction [31], and zenith wet delay is estimated as random walk noise process
Receiver position	Estimated as random walk process
Receiver clock offset	Estimated as white noise
Inter-system bias	Estimated as white noise
Ambiguities	Estimated as constants

**Table 2:** RMS Statistics of the PPP errors in the east, north and up directions.

	ELE	C/N0	ELE/CN0
East (m)	0.74	1.19	0.55
North (m)	0.75	0.77	0.73
Up (m)	1.83	1.62	1.35
3D (m)	2.11	2.15	1.63

**Table 3:** RMS statistics of the PPP errors in the east, north and up directions.

	ELE	CN0	ELE/CN0
East (m)	1.24	0.83	1.00
North (m)	0.71	1.28	0.62
Up (m)	5.03	3.03	2.22
3D (m)	5.23	3.39	2.51
